# “When It’s a Girl, They Have a Chance to Have Sex With Them. When It’s a Boy…They Have Been Known to Rape Them”: Perceptions of United Nations Peacekeeper-Perpetrated Sexual Exploitation and Abuse Against Women/Girls Versus Men/Boys in Haiti

**DOI:** 10.3389/fsoc.2021.664294

**Published:** 2021-09-24

**Authors:** Susan A. Bartels, Carla King, Sabine Lee

**Affiliations:** ^1^ Department of Emergency Medicine, Queen’s University, Kingston, ON, Canada; ^2^ Department of Public Health Sciences, Queen’s University, Kingston, ON, Canada; ^3^ Department of History, The University of Birmingham, Birmingham, United Kingdom

**Keywords:** gender, haiti, peacekeeping, united nations, sexual exploitation and abuse, MINUSTAH

## Abstract

Peacekeeping missions have been marred by reports of sexual exploitation and abuse (SEA) against local community members. However, there is limited research on how SEA against women/girls versus men/boys is perceived in peacekeeping host societies. In 2017 we collected micro-narratives in Haiti and then conducted a thematic analysis to understand how peacekeeper-perpetrated SEA was perceived by local community members comparing SEA against women/girls versus SEA against men/boys. Both male and female participants used language which suggested the normalization, in Haitian society, of both transactional sex with and rape of women/girls by UN personnel. In contrast, peacekeeper-perpetrated SEA against men/boys was viewed as unacceptable and was associated with homosexuality and related stigmatization. Overall, our results suggest that in Haiti, inequitable gender norms, the commodification of female sexuality, and homophobia result in SEA against males being recognized as a wrong that elicits outrage, while SEA against women/girls has been normalized. It is important to address the normalization of SEA against women/girls to prevent future violence and to recognize that SEA is also perpetrated against men/boys. Survivor-centered programs, sensitive to the needs of both male and female survivors, are required.

## Introduction

Both women/girls and men/boys experience sexual exploitation and abuse (SEA) perpetrated by peacekeeping personnel. However, little research has been conducted on how local host communities perceive SEA against each of the sexes and what implications those perceptions might have for the response to peacekeeper-perpetrated SEA. To address this gap, our current work analyzes cross-sectional data from Haiti to explore local community members’ perceptions about SEA perpetrated against women/girls versus SEA perpetrated against men/boys by members of the United Nations Stabilization Mission in Haiti (MINUSTAH). The objective of this research is to understand how SEA is conceptualized in Haiti based on the victim’s gender and to identify implications of this conceptualization on the prevention and reporting of and responses to, SEA. We begin with an overview of the literature about gender norms in Haiti, sexual violence and peacekeeper-perpetrated SEA, as well as community perceptions of SEA, before outlining our methods. We then present mixed qualitative/quantitative results highlighting how SEA against women/girls was perceived differently from SEA against men/boys. The article closes with a discussion around the implications of the results and a number of recommendations.

### Critical Feminist Perspectives on Sexual Exploitation and Abuse in the Global South

Alongside many feminist scholars this project explores not the rape itself as “sexed” but takes the position as a starting point that rape “functions as a violent means of sexual differentiation; that is a society-wide means of producing and perpetuating a system of oppression that privileges heterosexual men” (Henderson, 2007). In the tradition of Bevequa (2000) analysis of the feminist struggles and in particular the nuanced analysis of the place of rape as part of a wider feminist political agenda (Bevacqua, 2000), the work moves on from the notion that rape has an ontological reality (Hulsman, 1986) and from the assumption that sexual crimes have an *a priori* existence that can be understood independently of the socially or economically specific context within which they occur. Instead, we specifically depart from the focus of victims of male violence as a phenomenon of the Global North, and we speak to Carrington, Hogg and Sozzo’s (Carrington et al., 2016) as well as DeKeseredy and Sanchez (DeKeseredy and Hall-Sanchez, 2018) efforts of decolonizing and democratizing the research agenda by foregrounding specific forms and patterns shaped by diverse cultural, social, religious and political factors in the Global South and particularly in Haiti. In doing so, we recognize the political dimension of feminist and critical theory (Kirby, 2013); but we also acknowledge that this research follows the pattern of much of the global knowledge economy, referred to as “extraversion” by Hountondji and Hountondji (1997), with theory and methodology often produced in the metropole and empirical data provided in and by the periphery (Roberts and Connell, 2016). This research, however, aims to highlight the contextualized experiences of local Haitian (female and male) victims of SEA and to critically interrogate how these voices contribute to advancing the “emancipatory potential” and “transformative alliance” aimed at challenging more effectively the power structures that sustain inequalities (Lykes and Moane, 2009).

### Gender Norms in Haiti

Politics and the local economy have helped to define gendered norms in Haitian society including “acceptable” roles for both women and men (Rames et al., 2016). Women’s gendered responsibilities often include unpaid work such as caring for children and managing the household (e.g., cooking and collecting firewood). In fact, women comprise more than 75% of Haiti’s informal economy (Maguire, 2012). In Haiti, when women are paid for their work, they earn on average 32% less than men, and the unemployment rate is twice as high for women as it is for men (World Bank Observatoire N, 2014). In rural regions the disparity is even more pronounced, with women almost three times more likely to be without formal employment than men, and most working in subsistence farming. Although the proportion of Haitian women engaged in paid labor is lower, their economic activities are key contributors to the purchasing power of their households (Rames et al., 2016) and women have continued to be “poto mitan”, or the center-posts of their households and communities (Maguire, 2012). Such a highly gendered division of household and formal labor incentivizes many Haitian females to partner with men who are employed and skilled (Kolbe, 2015).

Sexual relationships in Haiti tend to be heteronormative and patriarchal. Polygamy is common among Haitian men who often maintain concurrent partners, with common-law relationships being more prevalent than religious unions and legal marriages (Clark, 2006). Patriarchy normalizes sexual violence within relationships, with consent for sexual relations often implied in dating contexts (Faedi, 2008; Kolbe, 2015). As a result, the sexuality of adolescent girls is often closely monitored by family members who may encourage or discourage dating depending on the family’s socioeconomic status. In settings of extreme poverty, families sometimes encourage dating and transactional sex as a means of alleviating financial strain (Kolbe, 2015). Haitian women and girls are often financially dependent on men, and in many respects sexual norms compound gendered economic norms, commodifying sexual activity in much of Haitian society (Kolbe, 2015).

### Sexual Violence

Approximately one in eight Haitian women aged 15 to 49 have had a lifetime experience of sexual violence (l'Enfance and Program, 2018). A majority of female survivors who present for care post-assault report significant physical and psychological trauma, including the lack of an emotional response to the trauma, fear, and flashbacks (Deschamps et al., 2019). However, socioeconomic and environmental factors make seeking care difficult, and women assaulted by an acquaintance as well as younger victims are more likely to delay medical treatment (Deschamps et al., 2019). While Haitian law recognizes rape as a criminal act, insidious forms of sexual violence such as sexual harassment and intimate partner violence are not currently recognized as criminal acts. In Haiti, intimate partner violence and domestic violence are viewed as private matters (Clark, 2006), and date rape has not been legally recognized (Human Rights Watch, 2019). However, criminal code reform submitted in 2017 will address some of these gaps (Kapur and Muddell, 2016). Outside of legal marriage, sexual violence is punishable under general assault charges although underreporting is common and the judicial system has generally been ineffective at dealing with sexual assault allegations (Clark, 2006).

While sexual violence against women/girl is known to be under-reported, this is likely even more true for sexual violence against men/boys, which has been touted a “human rights violation that is too taboo to talk about” (Gorris, 2015). From a legal and policy perspective, male survivors of sexual violence have been referred to as “invisible victims” (Krug et al., 2002), and the World Health Organization (WHO) identified sexual violence against men/boys as a “significant problem” that had been “largely neglected” (All Survivors Project, 2021). More recently, however, increasing attention has shed light on the prevalence and consequences of sexual violence against men/boys, in both civilian (1 in 6, 2020; Sumner et al., 2016) and conflict settings (Christian et al., 2012; Solangon and Patel, 2012; Omona, 2014; Ferrales et al., 2016; Sumner et al., 2016; Human Rights Council, 2018). Consequences of sexual violence against men/boys are recognized and include sexually transmitted infections, genital and rectal trauma, sexual dysfunction, and urinary problems (Sivakumaran, 2007) as well as posttraumatic stress disorder, depression and anxiety (Martin, 2019). Many male victims also experience shame and humiliation, anger, fear and powerlessness, as well as a destruction of gender identity and confusion of sexual orientation (Solangon and Patel, 2012).

### Gendered Community Perceptions of Sexual Exploitation and Abuse in Peacekeeping Economies

It is well understood that social norms, particularly in rural contexts, reinforce sexual violence against women and children (Ricardo and Barker, 2008; Linos et al., 2013; Abeid et al., 2014; Thulin et al., 2020). In contrast, despite some early work on the gender stereotypes in reactions to victims (Howard, 1984), research examining male rape victims’ experiences and community perceptions of male rape has largely been missing from the discourse (Josenhansa et al., 2020). Similarly, although peacekeeping economies have been studied in some detail (Higate and Henry, 2004; Jennings and Nikolić-Ristanović, 2009; Henry, 2015), and their gendered nature has been discussed (Aning and Edu-Afful, 2013; Beber et al., 2019), local perceptions as they relate to male and female victims have not received any attention.

### Sexual Exploitation and Abuse by United Nations Peacekeeping Personnel

As a result of political instability and insecurity in Haiti, the United Nations sanctioned the peace support operation (PSO), MINUSTAH (United Nations, 2015). Operational from 2004 to 2017, the PSO was initially mandated to protect civilians from organized crime and armed gangs. However, following the 2010 earthquake, the UN expanded MINUSTAH’s mandate to support recovery, reconstruction and stability efforts in the country (United Nations, 2015). Among PSOs, MINUSTAH was particularly controversial (Wisner, 2015). The cholera epidemic which affected more than 800,000 Haitians and killed at least 10,000 (Houghton and Norris, 2018; Katz, 2016; Luquero et al., 2010-2011), was inadvertently introduced by UN peacekeepers in late 2010. Additionally, and more relevant to the current work, MINUSTAH has been at the center of extensive allegations of sexual exploitation and abuse (SEA) perpetrated against local community members. (Bracken, 2014; Shingler, 2016; Snyder, 2017; Lee and Bartels, 2019; Vahedi et al., 2019; King et al., 2020). These human rights abuses have included rape, sex with minors and human trafficking. Even when allegations of SEA have been investigated, justice has been lacking. In one example reported by the Associated Press, 134 Sri Lankan peacekeepers were known to have sexually exploited nine children in a sex ring (Snyder, 2017). Although 114 of the peacekeepers were repatriated only a handful ever faced any punitive action in Sri Lanka (REDRESS, 2020). Perpetration of SEA by peacekeeping personnel is not unique to Haiti. Over the years many PSO have been implicated in SEA scandals, including missions in the Balkans (Gustafsson, 2005; Simic, 2012), Liberia (Okigbo et al., 2014; Beber et al., 2017), the Central Africa Republic (Deschamps et al., 2015; Schechner and Hinshaw, 2015), the Democratic Republic of the Congo (IRIN News, 2005; News (2005). French, 2005; Notar, 2006), and Timor-Leste (Barker, 2006; Westendorf, 2020).

Deployed peacekeepers are imbedded into the geopolitics and prevailing ideologies of the host country and they engage with the existing socio-economic and gender norms (Autesserre, 2014). Indeed, the presence of PSO may reshape or reinforce a host society’s norms (Jennings, 2014), and peacekeeping economies have been well described (Higate and Henry, 2004; Henry, 2015; Jennings and Bøås, 2015). It is not unexpected therefore, that the arrival of foreign, predominantly male, well-paid peacekeepers would amplify gendered labor. In this way, the masculine nature of PSO augments the more informal and illicit work often made available to women in peacekeeping economies (waitressing, domestic labor, transactional sex, sex work) (Higate, 2007), thereby perpetuating the precarity of women’s work and reinforcing gendered divisions of labor (Jennings and Nikolić-Ristanović, 2009; Jennings, 2014).

In 2003, the UN introduced its “zero-tolerance policy” which states that sexual relationships between UN staff and beneficiaries of assistance are based upon “inherently unequal power dynamics” and are therefore “strongly discouraged” (United Nations Secretary, 2017). However, enforcement of the zero-tolerance policy has proven problematic for a variety of reasons. For instance, the *Status-of-Forces Agreements* (SOFA), which regulate the relationship between the UN, troop and police contributing countries (TPCC) and host countries, grants military peacekeepers functional immunity from host state jurisdictions for criminal acts undertaken during official duties (Simm, 2013). Disciplinary action over national military contingents is the responsibility of the TPCC (Ndulo, 2009; Blau, 2016) but member states are not legally obligated to prosecute even in cases of serious crimes such as rape and sex with minors (Rehn and Johnson Sirleaf, 2002).

Despite research describing peacekeeping economies, evidence on how PSOs engage with local identities and norms, as well as scholarship examining peacekeeper immunity and the environment of impunity, host community understandings and perceptions of UN peacekeeper-perpetrated SEA remain understudied. In particular, empirical work so far has focused on female victims (SenseMaker, 2017; Vahedi et al., 2019; King et al., 2020) and thus there is no empirical research examining how SEA against women/girls is perceived versus that perpetrated against men/boys. We undertook the current analysis to address this evidence gap and make recommendations around the prevention of and response to peacekeeper-perpetrated SEA.

## Methods

Through a cross-sectional study in June to August 2017, we collected self-interpreted micro-narratives from a broad range of Haitian community members. Full details of data collection have been described previously (Lee and Bartels, 2019) but will be summarized below. From the larger primary dataset, we currently present a thematic analysis of how SEA perpetrated against local women/girls was perceived differently than SEA perpetrated against local men/boys.

### Interview Tool

SenseMaker is a narrative-based capture tool that extracts meaning from shared micro-narratives, which has been found to be a valuable way for people to share complex information about their experiences (SenseMaker, 2017). We used SenseMaker to better understand interactions between Haitian community members and MINUSTAH peacekeeping personnel. Participants were requested to share a brief story in response to their choice of three prompting questions, all of which essentially asked for an anonymous narrative about their experiences of women/girls living in that particular community which hosted a PSO (included in the Supplementary Materials), which forms the qualitative part of the dataset. If the prompting question triggered participants to share a story about men/boys rather than women/girls, research assistants proceeded with the survey.

After audio-recording their micro-narratives, participants interpreted the experiences shared by responding to a series of pre-defined SenseMaker questions (included in the Supplementary Materials). This data was described in the primary study (Lee and Bartels, 2019) and is not included here. At the end, participants answered a series of multiple-choice questions on the nationality and position of the peacekeeper involved in the story as well as personal demographics. The responses to the multiple choice questions form the quantitative part of the dataset.

The SenseMaker survey was drafted with collective expertise in sexual and gender-based violence, humanitarian crises, public health, Haitian law and SenseMaker methodology, and it’s design included input from all three local partners. The survey was originally written in English, translated to Kreyòl and then back-translated to confirm accuracy. Discrepancies were resolved by consensus. A draft survey was pilot tested in Haiti with 54 participants and then refined based on those results and feedback.

### Study Implementation

We surveyed around 10 UN bases across seven locations in Haiti (Port-a-Prince, Léogâne, Port Salut, Hinche, Saint Marc, Cap-Haïtien, and Morne Cassé). Within a 30 km radius of each selected base, trained Haitian research assistants approached a convenience sample of prospective participants in naturalistic settings such as public transportation stops/depots, shops, and markets. Any male or female community member over the age of 11 was eligible to participate. There were no additional inclusion criteria, and it was not necessary to have had a personal experience of peacekeeper-perpetrated SEA in order to take part. Twelve Kreyòl-speaking research assistants were purposely selected from two of the local partners, Komisyon Fanm Viktim pou Viktim (KOFAVIV) and Enstiti Travay Sosyal ak Syans Sosyal (ETS). The six female and four male ETS research assistants were undergraduate students, and the two female KOFAVIV research assistants were volunteers with the organization who had expertise supporting clients of gender-based violence. Study implementation began with a 4-day team training that included SenseMaker methodology, informed consent, research ethics, a detailed question-by-question review of the survey, data upload, as well as management of adverse events and program referrals. All interviews were conducted in Haitian Kreyòl using iPad Mini 4’s. Native Kreyòl speakers transcribed and translated the transcripts from Kreyòl to English.

### Definitions

As per the UN definition, “sexual exploitation” was taken to be “any actual or attempted abuse of a position of vulnerability, differential power, or trust, for sexual purposes, including but not limited to, threatening or profiting monetarily, socially, or politically from the sexual exploitation of another” (United Nations, 2017). Sexual exploitation includes transactional sex and solicitation of transactional sex as well as exploitative relationships more broadly. “Sexual abuse” was used to refer to “the actual or threatened physical intrusion of a sexual nature, whether by force or under unequal or coercive conditions” (United Nations, 2017). All sexual activity with a child under the age of 18 was considered sexual abuse regardless of the age of majority or consent locally. Any individual, described as being under the care of parents/guardians, referred to as “girl” or “boy”, or referenced to be in school was assumed to be under the age of 18. For transactional sex, we used the definition of Stoebenau et al. as the non-commercial, non-marital sexual relationships motivated by the implicit assumption that sex will be exchanged for material support or other benefits (Stoebenau et al., 2016; United Nations, 2017). Adapted from the Wisconsin Coalition Against Sexual Assault, for the purposes of this analysis, we define “normalization” of SEA to mean the acceptance of SEA is an immutable part of life, that depictions of SEA do not have real life consequences, and that it is the responsibility of the victim, rather than the perpetrator, to prevent SEA (Wcasa, 2020).

### Analysis

**FIGURE 1:**
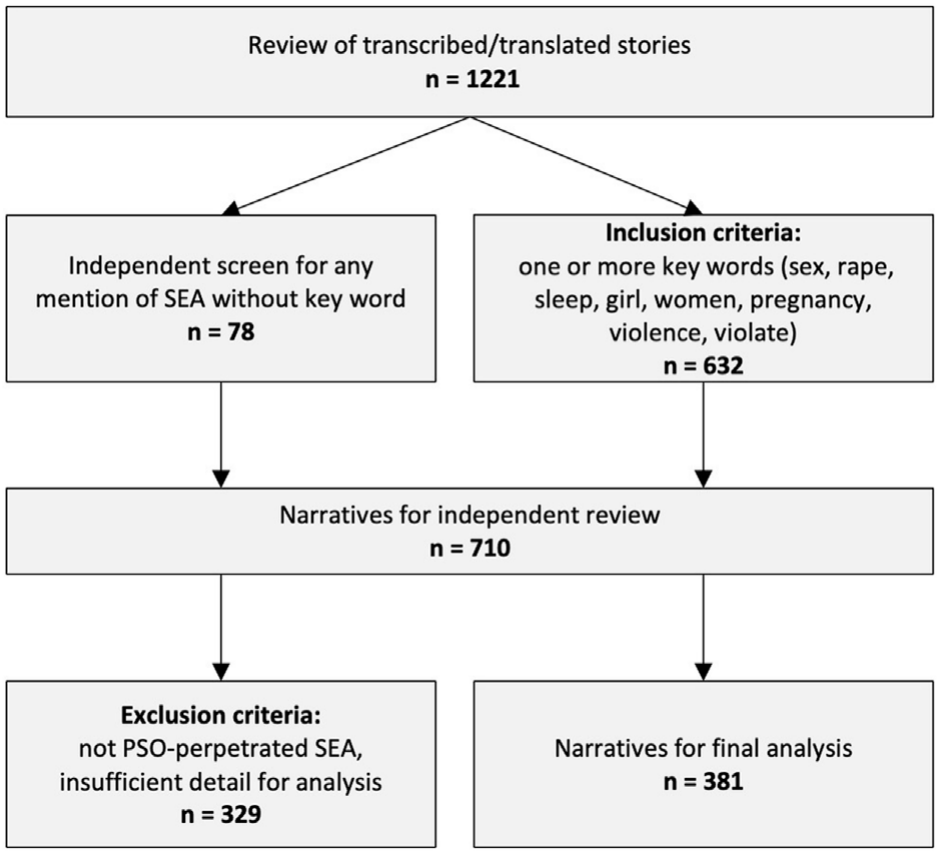
Narrative sampling process.

As described previously, we conducted a keyword search of the 1,221 transcribed and translated narratives (King et al., 2020). Keywords included: violence, violate, sex, rape, sleep, girl, women, and pregnant, providing a subsample of 632 stories. Another member of the research team had separately screened all 1,221 transcripts for any mention of sexual interactions, which added another 78 stories. Two co-authors (CK and SB) independently screened each of the resulting 710 narratives using the following inclusion criteria: any story about or mentioning sexual interactions between local male or female community members and UN peacekeeping personnel. Stories containing only a single phrase about sexual interactions and lacking any detail that would make it useful for qualitative analysis were excluded, yielding a final sample of 381 narratives as shown in Figure 1.

We conducted a thematic analysis using inductive coding and latent theming (Braun and Clarke, 2006). First order codes were created after familiarization with the data and two researchers (CK and SB) independently coded the narratives in Dedoose 8.2.14. According to Saldaña, line-by-line coding identified the diverse experiences and perceptions in each participant’s shared narrative (Saldaña, 2012). A pooled Cohen’s kappa (0.82) was used to determine coding inter-rater reliability. Codes were then organized into conceptual themes to understand perceptions how SEA differed according to the victim’s gender. Critical dialogue and triangulation between the researchers (CK, SL, SB) was maintained during analysis, and we were sensitive to the literature base. Additional insights were recorded through memoing and constant comparison helped to ensure that each story was considered in relation to other stories. An audit trail of codes and memos was maintained.

Using SAS^®^ Studio release 3.8 (Enterprise Edition), chi-squared tests detected significant associations (*p*-value less than 0.05) between themes and participant demographics (Fisher’s exact test was used when any cell count was less than 5). Age and education categories were collapsed to make more meaningful comparisons between groups.

### Ethics

No identifying information was collected. Research assistants reviewed the informed consent in Kreyòl and participants indicated their consent to participate by tapping a consent box on the tablet. Written consent was waived. Adolescents aged 11 and older were included because they were known to be affected by SEA, and it would have been unethical to exclude them. Parental consent was not sought since the girls were considered mature minors and involving parents would likely have introduced bias and risk for parental conflict and/or abuse (American Psychology Assoc, 2018). Because there were no questions about SEA or about sexual interactions, participants could choose to talk about whatever experience they wanted, and it was deemed acceptable to include younger adolescents. Additionally, KOFAVIV team members were onsite to offer immediate psychological support to participants, and all participants were offered referrals to KOFAVIV for further psychological services as well as to Bureau des Avocats Internationaux for legal counsel. Financial or other compensation was not offered. Our study protocol (# 6020398) was approved by the Queen’s University Health Sciences and Affiliated Teaching Hospitals Research Ethics Board.

## Results

Participants included in the analysis were predominantly male (71.9%), under the age of 35 (71.7%), and had a partial or complete secondary education (59.6%). As illustrated in Table 1, a majority had average household income (60.6%) and almost half were from Port Salut (28.1%) or Cité Soleil (19.2%).

**TABLE 1:** Participant demographics.

Characteristic		Frequency (%) *n* = 381
**Location**	Port Salut	107 (28.1%)
	Cité Soleil	73 (19.2%)
	Hinche	60 (15.8%)
	Saint-Marc	50 (13.1%)
	Cap-Haïtien	32 (8.4%)
	Léogâne	27 (7.1%)
	Charlie Log base/Tabarre	22 (5.8%)
	Morne Cassé/Fort Liberté	10 (2.6%)
**Sex**	Male	274 (71.9%)
	Female	105 (27.6%)
	Prefer not to say	2 (0.5%)
**Age (years)**	11–17	26 (6.8%)
	18–24	100 (26.3%)
	25–34	147 (38.6%)
	35–44	54 (14.2%)
	45–54	33 (8.7%)
	>55	13 (3.4%)
	Prefer not to say	8 (2.1%)
**Household Income**	Poor	108 (28.4%)
	Average	231 (60.6%)
	Well-off	42 (11.0%)
**Education**	No formal education	11 (2.9%)
	Some primary school	26 (6.8%)
	Completed primary school	43 (11.3%)
	Some secondary school	157 (41.2%)
	Completed secondary school	70 (18.4%)
	Some post-secondary school	53 (13.9%)
	Completed post-secondary school	21 (5.5%)

Thematic analysis of the 381 micro-narratives identified different perceptions of SEA against female community members versus SEA against male community members, with distinct language being used for each. These differences are explored in detail below along with a selection of illustrative quotes for each and suggest community members perceive SEA against women/girls as more normalized and accepted in comparison to SEA against men/boys.

### Sexual Exploitation and Abuse Against Female Community Members

The narratives suggest widespread acceptance that women/girls rely on transactional sex to meet their basic needs, increase their life opportunities, and improve their social status.

Transactional sex with women/girls was identified in 40.2% of all stories and was significantly more likely to involve Brazilian troops (*X* ^*2*^ (1, *n* = 381) = 11.97 *p* = 0.005) at Cité Soleil (*X* ^*2*^(1, *n* = 381) = 11.35 *p* = 0.001) compared to all other TPCC and locations, respectively. Participants under 35 years old were more likely to describe transactional sex than participants 35 years old and older (*X* ^*2*^(1, *n* = 381) = 4.56 *p* = 0.003).

Participants recognized the economic situation for women/girls in Haiti as a driving factor for such sexual interactions with UN peacekeeping personnel.

Based on the economic situation of the ladies, they had relationships. I can even say sexual with the white men because they got some things from the white men just so they can survive, buy what they need.

Single female, aged 18-24 describing unidentified MINUSTAH soldiers in Hinche.

In another example, a male participant described women selling their bodies out of “misery and hunger”. He goes on to say “[Women] have to use this way so they can make some money, so they can get some food.” (Married male, aged 35–44 describing Brazilian MINUSTAH soldiers in Cité Soleil).

Participants also noted, as in the following story, the power imbalance between Haitian women/girls and MINUSTAH personnel.

…they offer the girls money so they can have sex with them and you know how difficult it is in Haiti, if you offer the girls money it is hard for them not to be seduced. They feed them sometimes... they are the authorities, and they have money, they have power, and they do what they please.

Single male, aged 25–34 describing Chilean MINUSTAH soldiers in Morne Cassé/Fort Liberté.

In addition, 10.5% of stories about transactional sex described women’s motivations to exchange sex with a foreign partner as a means of improving social status, accessing desirable items (i.e. clothes, trips, gifts) or out of a desire to have a “beautiful child”. There were significant correlations between stories about a woman’s desire for a foreign partner and a narrator’s age (*X* ^*2*^(1, *n* = 373) = 4.73, *p* = 0.030) and marital status (*X* ^*2*^(1, *n* = 377) = 5.08, *p* = 0.020). Participants describing a woman’s desire for a foreign partner were more likely to be under age 35 and single/never married.

In the example below, the participant referred to “children” agreeing to have sex with Brazilian soldiers to improve their social status and economic situation, in addition to securing food.

… they have come to think that entering into a relationship with a foreigner could be a thing that can make it easier to eat, either to help themselves, or to get some chances, to find some way, to find a way to make themselves look good.

Single male, aged 25-34 describing Brazilian MINUSTAH soldiers in Hinche.

In another example, a female participant described MINUSTAH personnel giving “us money to pay for transportation to the beach … money for clothes.” She goes on to describe MINUSTAH “coming to live without condoms” (single female, aged 18–24 describing Brazilian MINUSTAH soldiers in Cité Soleil). “Live” is used in Haiti to describe sexual intercourse.

Perceptions of women with a desire to have beautiful children with a foreign partner may also have been embedded within transactional sex, since having children with a MINUSTAH peacekeeper could be an avenue through which women/girls secured ongoing relationships with their foreign partners and the economic supports associated with it.

I often see that they would like to make a child with MINUSTAH in order to have beautiful kids and some would say also that they like to be with a MINUSTAH because when she has a relationship with him, he used to give American money to her.

Single male, aged 25–34 describing Nepalese MINUSTAH soldiers in Port-au-Prince.

A participant in Léogâne provided another example of the perspective that women engage in transactional sex for improved social status and should be held responsible for having children with MINUSTAH soldiers.

But there are some of these girls who wanted to have and conceive children with MINUSTAH because they wanted these children to be beautiful, etc … As I understand it, it is not their fault but the fault of these girls who wanted at all costs to have children with MINUSTAH.

Married female, aged 25-34 describing unidentified MINUSTAH soldiers in Léogâne.

With the exception of the shaming noted above, participants appeared to accept that women engaged in transactional sex with UN peacekeepers and thus often did not recognize the abuse of power at the hands of MINUSTAH personnel. The following participant provided one example, whereby what started as sexual exploitation was only regarded as problematic when several Brazilian soldiers raped the women without paying.

Now, what the MINUSTAH officer did, he wasn’t satisfied with simply sleeping with her and paying her, you see what I am saying? He took 2 to 3 others, they grabbed her, the 2 others didn’t pay her.

Single male, aged 25–34 describing Brazilian MINUSTAH soldiers in Cité Soleil.

Similarly, for some participants, sexual interactions between MINUSTAH personnel and local women/girls were perceived negatively only when there was no payment or when the payment was perceived to be inadequate. This opinion was shared by both male and female participants. In the following story, a participant expressed his disapproval of peacekeepers engaging sexually with Haitian women without paying them “a gourde”.

When I left Gonaives, I returned to Saint-Marc, it was the same thing. It was the same thing, sleep with young women, without giving them a gourde.

Single male, aged 25–34 describing Uruguayan MINUSTAH police in Saint-Marc.

Some participants perceived that sexual interactions were not exploitive because the women/girl had benefited from the interaction in one way or another. In these cases, there was a lack of appreciation that women’s constrained choices compromised their ability to consent for sexual interactions. A female participant from Cité Soleil reported, “They sometimes give them money to pay rent for their house,” and then concluded “I don’t see that as violence” in reference to local women having sex with peacekeepers (single female, aged 25–34 describing unidentified MINUSTAH personnel in Cité Soleil).

Other participants referred to UN peacekeepers as having the means to have sex with Haitian girls, implying that SEA of women/girls was permissible as long as there was monetary compensation. For instance, in the following story, this participant referred to the buying of schoolgirls’ virginity.

You can take the example of the young schoolgirls - maybe MINUSTAH takes their virginity because they have the means, they have the dollar bills.

Single male, aged 18-24 describing unidentified MINUSTAH soldiers in Hinche.

Additionally, some participants described sexual exploitation or abuse as accepted and even covered up, presumably so that the financial and/or material benefits derived from the sexual transactions could continue.

….But sometimes they do it by force by violating or seducing them … after being abused by these agents [they] did not want to denounce them. They have preferred to keep it secret for them.

Single male, aged 25–34 describing Brazilian MINUSTAH soldiers in Charlie Log Base/Tabarre.

Even when narratives referenced rape and sexual abuse of young girls, some participants specifically referred to victims “selling” their bodies, as if the blame lay wholly, or partially, with the girls themselves. Recognition that the girls were underage and therefore not able to give consent for sexual relations was often completely absent as illustrated by this participant.

They invade our homes and take our daughters away and rape them … These girls went and sold their bodies for five pennies, 1,000 gourdes, 100 dollars and 20 dollars.

Married male, aged 45–54 describing Brazilian MINUSTAH soldiers in Cap-Haïtien.

Other participants seemed to accept that when peacekeepers were deployed without their partners, SEA was inevitable. Additionally, some victims were referred to as having “weak minds”, although it is unclear if this referred to girls’ morality, intellectual abilities, or other circumstances.

Sometimes some of the guys do not have a wife. They can take two teenage girls and they take advantage of them, often the ones with weak minds.

Single male, aged 25-34 describing Brazilian MINUSTAH soldiers in Cité Soleil.

Even when MINUSTAH personnel were referred to as rapists, as in the following story, there was a sense that compensation would have righted the wrong.

The MINUSTAH left its mark as rapists. I can tell you that up to now, we did not get any compensation, that the person in question did not get the type of compensation she should.

Single male, aged 25–34 describing Uruguayan MINUSTAH soldiers in Port Salut.

It is unclear what type of compensation was being referenced but presumably financial or material. There was no mention of justice in the legal sense.

### Sexual Exploitation and Abuse Against Male Community Members

SEA perpetrated against men/boys was twice as likely to be categorized as rape (93.1%) in comparison to SEA against women/girls (46.6%). Transactional sex almost exclusively involved women/girls, with only 6.9% of SEA against men/boys describing an exchange of money, food or goods for sex. Male participants contributed 77.9% of stories about SEA against men/boys. These stories were strongly associated with Port Salut (*X* ^*2*^(7, *n* = 381) = 38.04, *p* < 0.001), likely as a result of the highly publicized rape of a local male youth in 2011 (Okigbo et al., 2014).

Language used to reference sex with men/boys and women/girls also differed, even in examples of non-descript sexual interactions and sex with minors. They often noted sex or pregnancy in relation to women/girls versus rape of men/boys.

You see what I’m saying because there have been a lot of children that go to ask them, you see what I’m saying? When it’s a girl, they have a chance to have sex with them. When it’s a boy, for example, they have been known to rape them.

Single participant, aged 25-34 describing unidentified MINUSTAH in Cité Soleil.

Despite a significant association between stories from Port Salut and rape of men/boys overall, stories from Cité Soleil were more likely to describe sexual interactions with the gender difference illustrated above (Fisher’s Exact Test, *p* = 0.007).

These narratives notably delineated between girls who were perceived to seek out MINUSTAH personnel to exchange sex for financial support versus boys who were perceived to be humiliated rape victims. This perspective was shared by both male and female participants.

We have young girls who think they are white... they think at least if they have a child for them [MINUSTAH], they would take care of the child. There are some who have two or three children for MINUSTAH.… We have young boys … MINUSTAH seized them and they sodomized them. This victimizes a person, humiliates and degrades him.

Married male, aged 25–34 describing Brazilian MINUSTAH personnel in Cité Soleil.

Some participants believed that MINUSTAH had introduced sexual violence against men/boys and that it had not existed in Haiti prior to arrival of the UN. Participants also, in some respects, felt that the rape of girls was inevitable, while the rape of boys was unexpected. For example, “You would think men would rape just girls. They also rape boys too” (single male, aged 25–34 describing unidentified MINUSTAH soldiers in Hinche).

Participants, as in the following example, voiced that sexual violence against men/boys was perceived as one of the things hated the most in Haiti. Similar language was not used when discussing sexual violence against women/girls.

MINUSTAH started to do things that are outside of its mission … they started to do one of the things we hate the most in Haiti. They started to commit rapes against boys.

Single female, aged 45–54 describing Uruguayan MINUSTAH soldiers in Port Salut.

Homosexuality was frequently mentioned in relation to SEA against Haitian men/boys and presented as an issue that MINUSTAH had introduced to Haiti (*n* = 35). Similar to stories of rape of men/boys, participants specifically noting homosexuality were more likely to be 35 years and older (*X* ^*2*^(1, *n* = 373) = 5.70, *p* = 0.024) and from Port Salut (*X* ^*2*^(7, *n* = 381) = 14.42, *p* = 0.044). The following male participant spoke of homosexuality as foreign and unwelcomed.

… in Haiti we do not have the system of homosexuality, we do not like it. However, the MINUSTAH had homosexual relations with and raped little X. We are asking that the United Nations personally compensate X and his family.

Married male, aged 35-44 describing Chilean MINUSTAH soldiers in Port Salut.

Again, there is reference to compensation and no mention of legal justice.

Homosexuality seemed to create fear among men as noted by one participant who stated that he was scared MINUSTAH would put their hands on him, “...when I see MINUSTAH, I run because I’m scared they will have homosexual acts with me” (single male, aged 18–24 describing Sri Lankan MINUSTAH soldiers in Léogâne).

A similar fear was not shared by women, or by men about women’s safety. However, there were references to children not being safe given the presence of MINUSTAH peacekeepers in the community (i.e., children being taken and abused).

The following participant specifically mentioned morality in reference to sexual violence against men/boys while there were no such references regarding SEA against women/girls. The participant clearly identified an issue of morality although it is unclear to what degree he was referencing homosexuality versus rape.

…we have children that are coming to see it, this case of homosexuality …. yes they are raping little boys - we are talking about morality.

Single male, aged 25–34 describing unidentified MINUSTAH soldiers in Cité Soleil.

Another participant gave the impression that SEA against men/boys was more intolerable and that when men/boys were being sexually assaulted, action had to be taken. For example, one participant reported:

Well, if it wasn’t for deputy X, there really would have been a lot of rape by the MINUSTAH around here. He had to kick them out because they started to commit homosexual acts on men. Women were raped, they also had children with some women and did not take care of them.

Married male, aged 25–34 describing Uruguayan MINUSTAH soldiers in Port Salut.

While the participant also makes note of peacekeepers fathering children and abandoning them, from his perspective, it was really the sexual violence against men/boys that prompted the deputy to act.

Participants used stigmatizing language (*n* = 20) more frequently in relation to male victims of SEA (*n* = 16) in comparison to female victims (*n* = 4). The following participant described Haiti as becoming dirty and misrepresented as a result of homosexuality initiated by MINUSTAH.

There was a young boy who came out of the closet and revealed on Facebook that he had been sexually assaulted by the MINUSTAH. Indeed, this problem for Haitians is almost unbearable and I am shameful as a citizen to see that the image of our nation is getting dirty and misrepresented.

Single male, aged 25-34 describing Uruguayan MINUSTAH soldiers in Hinche.

Another participant relegated boys who were sexually assaulted to “no longer have an existence in society.”

… children who were victims of sexual violence can’t really be part of the society anymore. Considering a boy 14, 15, 16 years old sexually assaulted by MINUSTAH agent who caught that on camera and published that on television, that boy no longer has an existence in society.

Single male, aged 25-34 describing Nepalese MINUSTAH soldiers in Saint Marc.

Narratives about shame experienced by men/boys was seemingly not limited to sexual assault; it also extended to transactional sex. For example, the following participant described both boys and girls engaging in transactional sex, but notes associated shame only for the boys.

The girls continue their sexual activities with the soldiers who offer them money and goods … After they convinced the boys to have sex with them in exchange for goods, the word got out and the boys involved in this behavior with the soldiers fled the area shamefully.

Single male, aged 11-17 describing unidentified MINUSTAH soldiers in Hinche.

In contrast to stigma experienced by male victims, participants centered SEA-related stigma for women/girls around reporting and pregnancy. For instance, in the following example, a participant describes a woman’s desire to hide her pregnancy to avoid related shame.

There are a lot of women who got pregnant by the MINUSTAH and they cannot reveal that because of shame, this is the culture of the country.

Single male, aged 18–24 describing Chilean MINUSTAH soldiers in Cap-Haïtien.

## Discussion

To the best of our knowledge, this case study in Haiti is the first analysis of empiric data to compare local perceptions about peacekeeper-perpetrated SEA against women/girls versus men/boys. Language used by participants highlight that SEA of local women/girls by UN personnel is normalized in Haitian society, particularly when it comes to transactional sex. Participants gave the impression of accepting SEA against women/girls as a part of economic necessity and female victims were more often held responsible for the consequences of their actions (i.e., pregnancy). In contrast, although SEA against local men/boys was less common, participants gave the impression of a much stronger negative reaction towards it, perceived that it was completely unacceptable, and expressed significant stigma. In Cité Soleil, for example, where transactional sex with women/girls was most frequently reported, participants were more likely to use normalizing, non-descript language to describe SEA of women/girls such as: “they had relationships”, “slept with young women”, “took advantage of them”, or “took their virginity”, while in contrast, men and boys were “raped.” These findings are particularly noteworthy among male participants who constituted the majority of the research participants.

Critical feminism with its creation of conceptual frameworks for understanding the construction of hegemonic masculinity has played a key role in uncovering and appreciating the nature of violence against both women and men. However, Brownmiller, in acknowledging the lack of visibility of male victims of sexual violence, suggests in her seminal work on rape that male victims are not actively “forgotten”, but female victims usurp them as the latter is “more acceptable” (Brownmiller, 1975). However, as the local perceptions of Haitian local communities demonstrates, both women and men are harmed by SEA, and different and gendered manifestations of shame, within the concept of hegemonic masculinity (Stanko and Hobdell, 1993; Walklate, 2004), may explain the gendered specificities of experiencing such abuse.

### Transactional Sex

With regards to MINUSTAH-perpetrated SEA against Haitian women/girls, our findings align with Stoebenau et al.‘s typology of transactional sex in Sub-Saharan Africa (Stoebenau et al., 2016). Within the “sex for basic needs” paradigm, women/girls were perceived as vulnerable victims left with little choice but to exchange sex for money, food, or other material support as a result of poverty and economic dependence on men. Transactional sex of this nature was evident in our data through stories of women experiencing “selling their bodies because of misery and hunger”, and exchanging sex for “some things from the white men just so they can survive”. In Stoebenau et al.‘s “sex for improved social status” paradigm, women/girls exchanged sex in response to relative deprivation in the setting of a growing consumer culture and desire to maintain a modern lifestyle, which is sometimes leveraged to increase social capital (Stoebenau et al., 2016). This was also evident in our research through stories of women’s desire to have “beautiful children” and using sex as a means to acquire money for clothes and transportation. Stoebenau et al.‘s “sex and material expressions of love” transactional sex paradigm highlights the centrality of gift exchange in romantic relationships, the idea that love and money are inextricably linked in romantic relationships, and the role of men as providers of material support (Stoebenau et al., 2016). Our narratives describing “relationships and dating” and of MINUSTAH “loving and taking care of” Haitian women support this paradigm. Little has been written about transactional sex in Haiti, but our findings align with that of Daniel and Logie who reported that material exchange including sex underlies many sexual relationships in Haiti and that the motivations behind transactional sex ranged from survival needs, to parental pressure to help support their household, to peer pressure to satisfy socio-emotional needs (Daniel and Logie, 2017).

### Perceptions Around Justice

While our survey did not ask specifically about perceptions of justice around sexual violence, multiple participants volunteered statements suggesting that injustice stemmed from the fact that no payment was offered to the women/girl or because payment was not considered sufficient. In these instances, there was no indication that the sexual act was viewed as exploitative or as an injustice but rather that payment was the concern. These findings are consistent with studies from the African context. For example, in Tanzania transactional sex was not considered exploitative if the male partner provided the expected payment or support (Wamoyi et al., 2019). There were also notable differences however, since participants in the Tanzanian study believed that transactional sex was exploitative when there was an imbalance of power based on age, economic capital, or social status. Research in Uganda similarly found that transactional sex was considered exploitative if it involved sex with a minor, misled a naïve or immature girl who lacked ability to give consent, or if there was a perceived power differential between the man and women/girl (Kyegombe et al., 2020). Our findings differ in this regard - there was little dialogue to suggest that exchanging sex with an underage girl was perceived as more exploitative or abusive than exchanging sex with an adult woman. However, our study was not designed to understand local perceptions of these power imbalances and we recommend this as an area for future study.

Narratives describing rape rather than transactional sex were also interesting from the perspective of perceptions around local justice. In our data, no individuals discussed justice in the legal or criminal sense of the word even in reference to rape of children. In fact, the only references to justice focused on economic compensation or reparation for the wrong committed. This is similar to the conceptualization of justice around sexual violence in the Democratic Republic of Congo (DRC) as published by Aroussi (2018). In her work, survivors of sexual violence were less interested in the prosecution of perpetrators and similar to our current work, no one mentioned criminal prosecution or legal justice. As Aroussi suggested, “reparation of the harm and restoration of the victim” may be more highly valued than retribution.

UN peacekeeping personnel have functional immunity from prosecution in the host country and can only be held accountable by the TPCC. The current results provide little insight, however, as to whether local knowledge of their functional immunity impacted perceptions of justice. In other words, we do not know to what extent participants were aware of the peacekeepers’ functional immunity and if/how this affected their conceptualization of justice. There have been well documented instances of SEA, such as the Sri Lankan peacekeepers who never faced criminal action for trafficking children (Snyder, 2017; REDRESS, 2020), which may have contributed to participants’ conceptualization of justice as reparation because of the environment of impunity. This theory would be supported by one participant in the current study who referenced MINUSTAH personnel saying, “they are the authorities and they have money, they have power and they do what they please.” If the foreign peacekeepers were perceived as immune from criminal prosecution and to be relatively wealthy, the equation of justice with reparation would make sense.

### Sexual Violence Against Men/Boys

The rape of men/boys was presented by participants in stark contrast to the normalized and somewhat accepted SEA of women/girls. Participants described affected men/boys as “victimized”, “humiliated”, and “degraded”. SEA involving men/boys was discussed as an issue of “morality” that was “unbearable and shameful”, and because of which MINUSTAH had to be “kicked out.” No such language was mentioned in reference to SEA of women/girls. This difference between perceptions of women/girls who experienced SEA versus men/boys who experienced SEA is likely multifactorial in origin - resulting from inequitable gender norms, the commodification of female sexuality, and a culture in which homosexuality is not widely accepted, as is the case in Haiti (Bracken, 2018). Since our study examined interactions between peacekeepers and local community members, we do not know whether sexual violence against men/boys perpetrated by non-UN personnel would elicit a similar reaction.

The stigma and shame associated with male survivors of sexual violence is consistent with other research, in which it has been theorized that the process of “homosexualization” through rape lowers a male victim’s “social status” by reducing him to a weaker symbolic identity (Sivakumaran, 2007). In settings of extreme homophobia, this can lead to severe stigmatization. Referring to a boy who had been sexually abused by a member of MINUSTAH, one participant said the victim “no longer has an existence in society”. While this may seem extreme, it is not unique to Haiti. In the DRC, the rape of men reportedly, “reduces the man to a useless being, he loses authority and his personality in the community” (Apperley, 2015). Many male survivors are hesitant to seek medical care and psychosocial services (Donne et al., 2018; De SasKropiwnicki Gruber et al., 2018; The Williams Institute, 2018; Ngari, 2016), putting them at risk of HIV seroconversion, related injuries and psychological distress.

### Limitations and Strengths

Although we attempted to collect narratives from a wide range of participants, the sample is not representative and thus the results cannot be generalized. Despite considerable effort to recruit women/girls, the final sample was two thirds male as many women/girls declined to participate. Local partners offered a number of possible explanations for the reluctance of females to participate including women not having enough time (e.g., due to childcare or domestic responsibilities) or women being less visible in the public spaces where recruitment occurred. In addition, women may have been unwilling to share personal traumatic or disturbing experiences, particularly rape, or uncomfortable talking about transactional sex for which they might fear being judged. Perceptions discussed considered the aggregate perceptions (i.e., not perceptions disaggregated by the research participants’ sex); therefore findings may be more representative of male perceptions. However, the disproportionate number of male participants does not, in our opinion, minimize the significance of the results since men constitute approximately 50% of the population and their acceptance/perceived normalization of SEA against women/girls is both problematic and worrisome. Second, SenseMaker narratives are shorter and less detailed than more traditional qualitative research, and the stories sometimes lacked the richness expected of in-depth interviews. Third, a more objective understanding about the nature of the sexual interactions between MINUSTAH personnel and Haitian men/boys as well as Haitian women/girls was lacking (i.e., it was impossible to know whether the sexual encounters constituted rape, exploitation, or had some degree of consent when two adults were concerned as we were entirely reliant on the narrator’s perspective). Finally, we are cognizant of our positionality and of the fact that as non-Haitian academics, the findings are interpreted with our own inherent biases.

The study also has several strengths that deserve mention. With a relatively large qualitative sample (*n* = 381), the data provide a wide range of perspectives from Haitian community members about SEA perpetrated by peacekeeping personnel. Because the accounts of SEA involved both men/boys as well as women/girls, we had a unique opportunity to understand how perceptions of SEA differed depending on whether the survivor was male or female. The narrative capture approach with its intentionally broad story prompts allowed the shared stories to flow naturally, thus contextualizing them within the participant’s broader lived experience.

### Recommendations

It is important to recognize that both males and females are being harmed by peacekeeper-perpetrated SEA, and our results are the first to highlight that they are being harmed in different and nuanced ways. In Haiti, inequitable gender norms, the commodification of female sexuality, and homophobia resulted in SEA against men/boys expressed as a wrong that elicited outrage. In contrast, SEA against women/girls was more widely accepted and the language used by participants suggested that it is normalized in Haitian society. The normalization of SEA against women/girls must be addressed so that future SEA can be prevented and reported to ensure that those affected receive needed care and services. However, it is equally important to recognize that SEA is also perpetrated against men/boys and they may experience qualitatively different experiences of stigmatization from the community. While service providers aim to provide survivor-centered programs, inclusivity is critical as is being sensitive to the fact that the needs of male survivors may differ from those of female survivors.

These findings lead to a number of recommendations. First, there is an urgent need for sensitization at the community level to increase recognition and awareness about what constitutes SEA, particularly as it relates to underage children. It is critical that host community members understand that children under the age of 18 cannot, under any circumstance, give consent to have sex with an adult, and importantly, this applies equally to boys and girls. It is also essential that this sensitization include information about how to recognize power imbalances which may compromise an adult’s ability to give consent for sexual relations as well as how to identify coerced sex. SEA is grossly under-reported and sensitization may be an important step towards increasing community members reporting of peacekeeper-perpetrated sexual misconduct. We would expect reporting of SEA against women/girls to be low if it was normalized in society, as was demonstrated in the current analysis. Reporting is clearly relevant from an accountability perspective, but it is also critically important from a health perspective to ensure those affected have access to medical care for STIs, HIV testing and treatment, pregnancy care, and safe abortion services as well as psychosocial support and mental health services.

Second, given the extreme poverty faced by many families in Haiti, sensitization alone will likely not lead to substantial change unless there is also investment in educational opportunities, income-generating activities, and small business loans so that individuals will have an option other than to accept SEA as the most feasible way to feed their families, meet their basic needs and access consumer goods. While the UN Trust Fund program does help to support women/girls already affected by peacekeeper-perpetrated SEA (United Nations Secretary, 2019), much broader systemic change is need in order to address high levels of existing poverty that render host community members, particularly women and girls, vulnerable to SEA.

Third, the current analysis raises several issues as priorities for future research. These include studies to provide a more comprehensive understanding of local perceptions around rights and justice as they pertain to SEA. SEA perpetrated by UN personnel is largely under-reported and this may occur partially because local conceptualizations of justice are at odds with how the UN, and the global north more broadly, conceptualize justice for sexual violence. Understanding how the two diverge or are discordant is important for improving reporting of SEA and also for re-aligning approaches to justice to be more sensitive and in tune with the needs of those affected. Finally, we recommend additional research to better understand the needs of men/boys affected by SEA, ensuring that they receive survivor-centered care and recognizing that their needs may, at times, be different from those of women/girls and unique approaches may be required.

## Data Availability

The raw data supporting the conclusions of this article will be made available by the authors, without undue reservation.

## Abbreviations

ETS, enstiti travay sosyal ak syans sosyal; KOFAVIV, komisyon fanm viktim pou viktim; MINUJUSTH UN, mission for justice support in haiti; MINUSTAH UN, mission for justice support in haiti; PSO, peace support operation; SEA, sexual exploitation and abuse; SOFA, status of forces agreement; TPCC, troop and police–contributing countries; UN, united nations.
