# Supplementary material for: “When It’s a Girl, They Have a Chance to Have Sex With Them. When It’s a Boy…They Have Been Known to Rape Them”: Perceptions of United Nations Peacekeeper-Perpetrated Sexual Exploitation and Abuse Against Women/Girls Versus Men/Boys in Haiti
Source: Front Sociol. 2021 Sep 24;6:664294. doi: 10.3389/fsoc.2021.664294 (PMC8498325; doi:10.3389/fsoc.2021.664294)
Supplement: Supplementary file 1 [file DataSheet1.docx]

**Supplementary Material - Survey questions with possible responses**

| **Question** | **Possible Responses** | |
| --- | --- | --- |
| Micro-narrative prompts | | |
| Describe the best or worst experience of a particular woman or girl in your community who has interacted with foreign UN or MINUSTAH personnel. What happened? | Micro-narrative recorded by participant | |
| Describe how living in a community with a UN or MINUSTAH presence has provided either a particular opportunity or a danger to a particular woman or girl in the community. What happened? | Micro-narrative recorded by participant | |
| Describe the negative or positive experience of a particular women or girl who requested support or assistance after interacting with foreign UN or MINUSTAH personnel. What happened? | Micro-narrative recorded by participant | |
| Dyads | | |
| The interaction and relations you shared in the story were… | 1) Entirely initiated by the foreign UN or MINUSTAH personnel;  2) entirely initiated by the woman / girl  or some combination thereof | |
| In the story you shared, who had power and control? | 1) Foreign UN or MINUSTAH personnel;  2) Woman / girl  or some combination thereof | |
| In relation to the woman or the girl in the story you shared, those in power… | 1) Did absolutely nothing to assist or support her;  2) Provided her with too much assistance and support  or some combination thereof | |
| Events in the story led the community to have an… | 1) Overwhelming desire to engage with the UN or MINUSTAH;  2) Absolute rejection of the UN or MINUSTAH or some combination thereof | |
| Triads | | |
| This story is about… | 1) Financial/material security;  2) Social status;  3) Emotional needs  or some combination thereof | |
| In this story, the foreign UN or MINUSTAH personnel was… | 1) In a position of authority;  2) Able to offer protection;  3) Wealthy and able to provide support  or some combination thereof | |
| Was the interaction in the story | 1) Friendly;  2) Business;  3) Relationship  or some combination thereof | |
| In the story, what would a fair response look like? | 1) Acceptance of responsibility;  2) Justice;  3) Reparation  or some combination thereof | |
| In the story, it would have helped the woman or girl most to have had support from… | 1) The UN or MINUSTAH  2) NGOs or civil society organizations  3) Haitian authorities  or some combination thereof | |
| In the story, barriers to the woman or girl getting a fair response were… | 1) Lack of information in the community about assistance;  2) Lack of response from Haitian authorities;  3) Lack of response from the UN or MINUSTAH  or some combination thereof | |
| In the story, what would have helped most to make the experience more positive? | 1) Material/financial support;  2) Emotional support;  3) Legal support  or some combination thereof | |
| Based on the events in the story, the presence of the UN or MINUSTAH led to… | 1) Disrespect of Haitian values and laws;  2) Negative financial impact;  3) Anger and resentment  or some combination thereof | |
| Multiple Choice Questions | | |
| Who is the story about? | | About me  About someone in my household  About someone in my family who doesn’t live in my household  About a friend  About someone else in my community  Something I heard or read about  Prefer not to say |
| How often does the situation in this story occur? | | Very rarely  Occasionally  Regularly  Very frequently  All the time  Not sure |
| How important is it for others to hear and learn from your story? | | Must hear this story and take action  Should definitely hear this story and pay attention  Can learn some things but not much  Nothing to learn from this story  Not sure |
| Who would most benefit from hearing the story shared (choose up to three)? | | Family  Friends  Neighbours  Haitian politicians  UN or MINUSTAH  NGOs  Military of the foreigner  Churches  Community leaders  Business people  Girls in my community  Women in my community  Men in my community  Not sure |
| What is the emotional tone of this story? | | Strongly positive  Positive  Neutral  Negative  Very negative  Not sure |
| How does your story make you feel (choose up to 3)? | | Angry  Disappointed  Embarrassed  Encouraged  Frustrated  Good  Happy  Hopeful  Indifferent  Relieved  Sad  Satisfied  Worried  Not sure |
| What country was the foreigner in the story from? | | Uruguay  Sri Lanka  Pakistan  Nepal  Argentina  Bolivia  Brazil  Chile  Peru  Indonesia  Jordan  Nigeria  Pakistan  Indonesia  Senegal  United States  France  Canada  Japan  China  Other  Don’t know |
| What was the role of the foreigner with the UN or MINUSTAH? | | Soldier (UNPOL, MINUSTAH, or Multinational Forces)  Civilian who works with the UN (doesn’t wear a uniform)  Police  Worked for an NGO rather than the UN or MINUSTAH  Other  Don’t know |
| What is your gender? | | Female  Male  Prefer not to say |
| How old are you? | | 11– 17years old  18– 24 years old  25 – 34 years old  35 – 44 years old  45 – 54 years old  > 55 years old |
| What is your marital status? | | Married or living together as if married  Divorced/Separated from spouse  Widowed  Single, never married  Prefer not to say |
| What is your highest educational qualification? | | No formal education  Some primary school  Completed primary school  Some secondary school  Completed secondary school  Some post-secondary school  Completed post-secondary school |
| I'll read you a list of 5 items that some people have at home. Please tell me which of these you or your household owns. Your household consists of people who sleep under the same roof and eat the same meals. Chose as many as your family has. | | Radio  Mobile phone  Refrigerator or freezer  Vehicle such as a truck, a car or a motorcycle  Generator, inverter or a sun panel that provides electricity to your home.  None of the above |
| Here are some questions about your life.  - In most ways my life is close to my ideal.  - The conditions of my life are excellent.  - I am satisfied with my life.  - So far I have gotten the important things I want in life.  - If I could live my life over, I would change almost nothing | | 7 - Strongly agree  6 – Agree  5 - Slightly agree  4 - Neither agree nor disagree  3 - Slightly disagree  2 – Disagree  1 - Strongly disagree |
| Is there anything else you would like to say? | | Free text |
| *Response was optional for all questions. | | |
